# Characterizing post-covid conditions in a Turkish cohort: A prospective study: Öykü Turunç

**DOI:** 10.1093/eurpub/ckac129.542

**Published:** 2022-10-25

**Authors:** AN Emecen, O Turunc, N Sivye, AF Suner, E Basoglu Sensoy, S Keskin, B Unal

**Affiliations:** Department of Public Health, Dokuz Eylul University, Faculty of Medicine, Izmir, Turkey

## Abstract

**Background:**

Investigating the people who suffer from post-COVID health conditions is necessary to accommodate the demand for accessing healthcare. This study aims to describe post-COVID health conditions within six months after diagnosis.

**Methods:**

This study was conducted at Dokuz Eylul University Hospital, a tertiary care hospital in Ä°zmir-Turkey. Participants aged ≥18 years who were diagnosed as SARS-CoV-2 RNA positive in the hospital from November 1st, 2020 to May 31st were interviewed by phone at one, three and six months after diagnosis. Symptom frequencies were stratified by demographic and clinical characteristics. The dependent variable was having post-COVID condition according to World Health Organization's definition. We estimated logistic regression models to identify associated factors for post-COVID condition in the patients who had symptoms at baseline.

**Results:**

A total of 5083 people completed the third month's interview. The prevalence of post-COVID condition was 21.8% (n = 1108). Tiredness/fatigue (10.2%), muscle or body aches (7.3%) and dyspnea/difficulty breathing (4.8%) were the most common symptoms. Older age (65-74 aged groups versus 18-24 aged group, odds ratio-OR:1.57, 95% confidence interval: 1.10-2.25), female gender (OR: 1.97, 1.71-2.28), bad economic status (OR: 1.44, 1.13-1.84), having more health conditions (≥3 conditions, OR: 1.82, 1.28-2.55), having more symptoms (>5 symptoms, OR: 2.59, 2.20-3.07) and hospitalization (intensive care unit, OR: 1.98, 1.13-3.37) were found to be associated with reporting of post-COVID condition.

**Conclusions:**

This study identifies the prevalence and risk factors for post-COVID conditions in a large cohort of patients. The results of the study would guide the healthcare organizations in the planning of post-COVID management strategies.

**Key messages:**

• The prevalence of post-COVID conditions was 21.8%. Older age, female gender, having more health conditions, disease severity in the acute phase and bad economic status were risk factors.

• Clinical management strategies and country-specific healthcare planning should be devised for the post-COVID condition burden.

